# Complementary time-lapse datasets of x-ray computed tomography and real-time strain mapping for an *ex-situ* study of non-crimp glass fibre composites under fatigue loading

**DOI:** 10.1016/j.dib.2021.107157

**Published:** 2021-05-18

**Authors:** Anuj Prajapati, Stuart Morse, Ali Chirazi, Timothy Burnett, Philip J. Withers

**Affiliations:** a Department of Materials, Henry Royce Institute for Advanced Materials, The University of Manchester, Manchester M13 9PL, UK; b Department of Materials, The University of Manchester, Manchester M13 9PL, UK; c Visualization Sciences Group, Thermo Fisher Scientific, Bordeaux 33800, France

**Keywords:** Non-crimp glass fibre composites, X-ray computed tomography, Digital image correlation, Fatigue, Correlative characterization

## Abstract

Data published in this paper corresponds to a time-lapse *ex-situ* experiment aimed at analyzing the tension-tension fatigue damage in non-crimp glass-epoxy composites by multi-scale x-ray computed tomography (XCT) of the damage features and their timeline. This is then correlated with the strain fields obtained through digital image correlation (DIC). The XCT - DIC datasets by is acquired by interrupting mechanical fatigue tests at three time-steps, after the material has undergone 0 cycles, 70,000 cycles, 80,000 cycles, and 120,000 cycles. This is one of the first multi-modally correlated datasets available for these types of non-crimp glass fibre composites, which explore the structure-property relationship in a time-dependent behavior. This dataset can be used to explore glass-fibre composites microstructure under a progressive damage scheme and can be used to test and train a plethora of image processing and analysis techniques. This dataset can also be used as an attempt to model the fatigue behavior of quasi-unidirectional non-crimp fibre composites by image-based simulations.

## Specifications Table

**Table:** 

Subject	Materials Science
Specific subject area	Non-crimp fabrics, fatigue damage, correlative characterization, strain mapping, time-lapse *ex-situ* imaging
Type of data	Image: X-ray computed tomography data Images: Real time strain mapping in RAW format and movies by digital image correlation.
How data were acquired	XCT Data Acquisition: Region of interest Zeiss VersaXRM 520 (time-lapse), Thermo Fisher Heliscan Mk1 (full-field). XCT Data Processing: Thermo Fisher Avizo DIC: LaVision Strainmaster 2D hardware and software.
Data format	XCT reconstructed data in 2D .tiff format, registered between time-steps. DIC data – raw image files in proprietary .vc7 format – associated parametric metadata as .exp, .ims, .attr, .xml, .scales, and .set formats. Real-time strain maps as movies in .mp4 format.
Parameters for data collection	Non-crimp composite of unidirectional E-glass fibre bundles stitched to backing bundles (90^o^) with a layup of [[0/90]/[90/0]s] with the backing bundles next to each other and none in the center, sandwiched between 100 μm thick layers of cross woven fabric on outer surface.
Description of data collection	The fatigue test was interrupted for *in-situ* DIC and *ex-situ* XCT at three points, where the sample was taken out of the hydraulic testing machine and put into the scanner, generating four datasets of XCT and DIC at 0, 70,000, 80,000 and 120,000 cycles, including the pristine starting DIC and full-field CT at 0.5 and 0 cycles, simultaneously.
Data source location	Henry Royce Institute, Department of Materials, The University of Manchester, Manchester, United Kingdom. M13 9PL Latitude: 53.467698 Longitude: −2.231499
Data accessibility	The data is publicly available online at: Observing the evolution of fatigue damage and associated strain fields in a correlative, multiscale 3D time-lapse study of quasi-unidirectional glass fibre composites [Data set]. Zenodo - http://doi.org/10.5281/zenodo.4541235
Related research article	Observing the evolution of fatigue damage and associated strain fields in a correlative, multiscale 3D time-lapse study of quasi-unidirectional glass fibre composites Anuj Prajapati et al., 2020 IOP Conf. Ser.: Mater. Sci. Eng. 942 012039 DOI: 10.1088/1757-899X/942/1/012039

## Value of the Data

• This is one of the first multi-modal, spatially, and temporally correlated datasets available for the study of non-crimp fabric composites under fatigue loading. This is the first dataset to link micro-damage to macro-behavior via x-ray tomography and digital image correlation.
• This data can be used by a variety of materials scientists especially the composites community who want to analyze dynamic micro-mechanical behavior of materials from correlative imaging and characterization [1].
• This dataset can be used to pinpoint dynamic fatigue damage in the bulk microstructure. This dataset can also be used to train and test existing and novel image analysis routines and algorithms for fibrous composites, owing to the high-resolution and good contrast XCT data.
• This dataset can also be used to derive new insights on correlating the DIC strain maps to material damage observed in the XCT volumes using new parameters, and can be used to verify and augment existing knowledge on such materials [2].
• The dataset can also be used to model dynamic fatigue behavior of non-crimp fibre composites by image-based simulations.

## 1 Data Description

The XCT datasets included in the zip file are of two types: a full-field scan which includes the whole gauge region of the sample, and a region of interest scan.

The full-field scan is taken only once at the beginning of the time-lapse study with no induced damage and is acquired in the Thermo Fisher Heliscan Mk1. The settings of the scan are 80 kV at a voxel size of 5.8 µm at the medium autofocus setting. This full field XCT scan is stored in the zip folder ‘Full_field_0_cycles.zip’ as a series of 2D .tiff files.

The region of interest (RoI) XCT scans are taken 4 times during the study at after 0, 70,000, 80,000 and 120,000 cycles. These acquired in the Zeiss Versa 520 with a pixel size of 0.7 µm at an exposure of 20 s per projection, accelerating voltage of 70 kV and a 4x optical magnification. These 4 scans are registered spatially to investigate the same region upon changing the scale. The region of interest scans are stored in the folders ‘0_cycles.zip’, ‘70,000_cycles.zip’, ‘80,000_cycles.zip’, and ‘120,000_cycles.zip’ – corresponding to the XCT scans taken at 0, 70,000, 80,000, and 120,000 cycles. These are also stored as a series of 2D .tiff files.

DIC acquisition is captured using LaVision Strainmaster software and hardware system with a black-and-white speckle pattern on the specimen gauge, and the correlation is carried out using a subset size of 31 pixels. The strain movies are calculated from a 0-strain reference image. The RAW DIC files are stored in the folder ‘DIC_RAW.zip’, which contain further folders for each of the 0.5, 70,000, 80,000, and 120,000 cycles. Each folder includes raw image files in proprietary .vc7 format, and associated parametric metadata in .exp, .ims, .attr, .xml, .scales, and .set formats. The .exp file is the experiment file which can be opened directly in the LaVision Strainmaster software and will import the rest of the associated data into the software system. The strain map movies are in .mp4 formats and are stored in the folder ‘DIC_strain_maps_movies.zip’ for each of the 0.5, 70,000, 80,000 and 120,000 cycles.

## 2 Experimental Design, Materials and Methods

The material is a non-crimp based glass-fibre composite of unidirectional E-glass fibre bundles stitched to backing bundles (90^o^) with a layup of [[0/90]/[90/0]s] with the backing bundles next to each other and none in the center, sandwiched between 100 µm thick layers of cross woven fabric on the outer surface.

The sample specimen geometry is described in [1], and is made after sticking the composite between two layers of tabbing orthogonal material and is then milled by a 80 mesh garnet waterjet abrasive.

The workflow [1] involving time-lapse x-ray CT and digital image correlation was used to identify the progression of damaged regions and their associated strain levels through a load-controlled fatigue test with 1% max strain, 4 Hz frequency, and an *R*-ratio of 0.1. The fatigue tests were interrupted for DIC (*in-situ*) and CT (*ex-situ)* at 3 intermediate stages in addition to the first pristine stage in the timeline. The sample was not tested to failure but rather fatigue cycling was stopped when around 10% stiffness was lost.

**Fig. 1:**
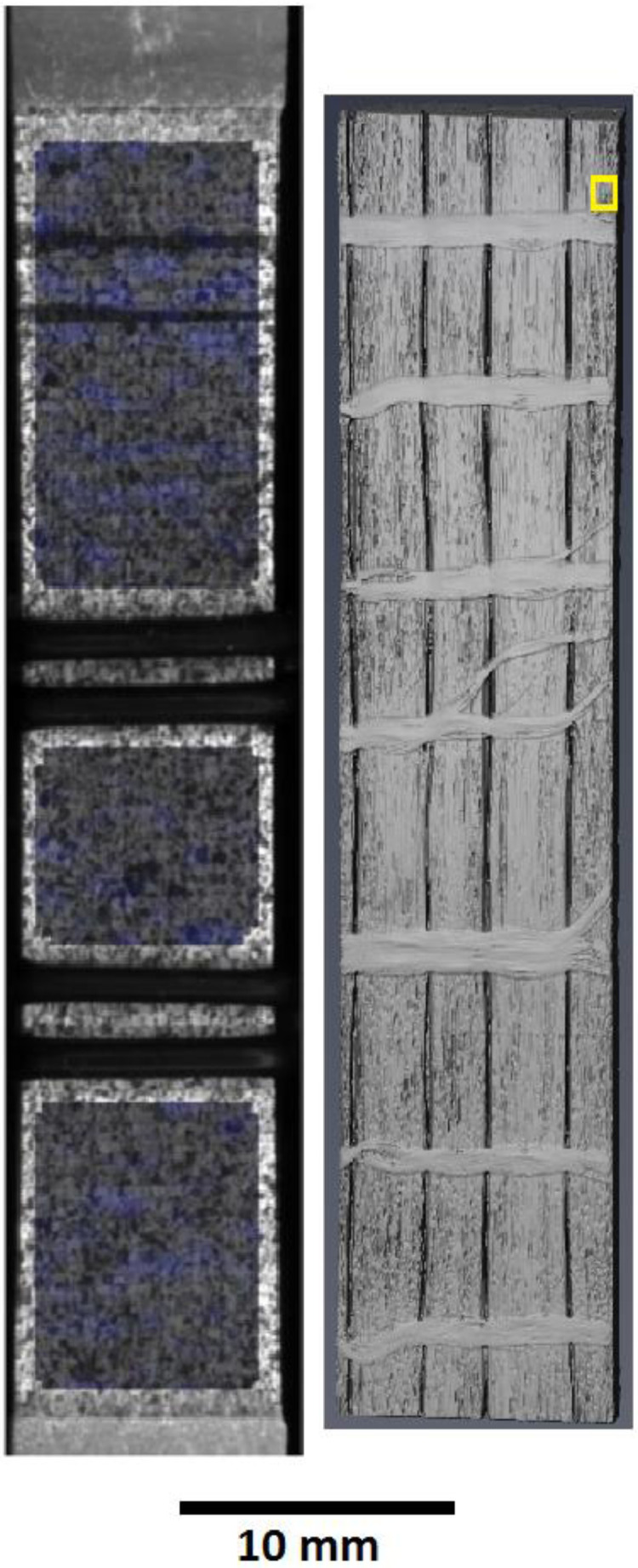
The yellow square on the right image near the top of the full-field XCT volume marks the region of interest for corresponding XCT scans. The left image shows the regions marked for DIC (For interpretation of the references to color in this figure legend, the reader is referred to the web version of this article.).

In the beginning, the sample is speckled using black and white spray paint on the surface opposite to the one where the knife-edges of the clip-on extensometer rest. This sample is then put through two XCT scans, the first being the full-field scan, which can be seen in Fig. 1 and is stored in the zip folder ‘Full_field_0_cycles.zip’ as a series of 2D .tiff files. The second scan is the higher resolution RoI scan which is taken from the region marked in Fig. 1 and is stored in the zip folders ‘0_cycles.zip’.

This sample is then taken to an Instron 8802 dynamic hydraulic machine, with a load cell of 100kN. It is then ramped up and down between 0 to 1% strain in load control mode, during which a DIC acquisition for the whole gauge length is acquired, corresponding to the ‘0.5cycles’ DIC and strain movie dataset. The sample is then sinusoidally cycled from 0.1% to 1% strain to 70000 cycles and another DIC acquisition is taken at the end with the same strain conditions, corresponding to ‘70000cycles’ DIC and strain movie dataset. This sample is then taken out of the hydraulic, XCT scanned in the same RoI using the same settings generating ‘70,000_cycles.zip’, and then brought back to the hydraulic to fatigue for another 10000 cycles, at the end of which another DIC acquisition is taken, corresponding to ‘80,000cycles’ DIC and strain movie dataset. This is then taken out of the hydraulic, XCT scanned in the same RoI using the same settings generating ‘80000_cycles.zip’, and then brought back to the hydraulic to fatigue for another 40,000 cycles, at the end of which another DIC acquisition is taken, corresponding to ‘120,000 cycles’ DIC and strain movie dataset. This sample then undergoes a final XCT scan in the same RoI using the same settings generating the ‘120,000_cycles.zip’.

All the XCT RoI data is spatially registered using Thermo Fisher's Avizo software using ‘Image registration wizard’. All DIC datasets are processed in LaVision Strainmaster software using the parameters of correlation mentioned above. The areas of the O-rings for extensometer placement and edges are excluded for DIC analysis, as can be seen in Fig. 1.

## CRediT Author Statement

**Anuj Prajapati:** Conceptualization, Methodology, Data curation, Software, Validation, Writing – original draft, Writing – review & editing; **Stuart Morse:** Data curation, Resources; **Ali Chirazi:** Conceptualization, Methodology, Software, Validation, Supervision, Writing – review & editing; **Timothy Burnett:** Conceptualization, Methodology, Writing – review & editing, Supervision, Funding acquisition; **Philip Withers:** Conceptualization, Methodology, Writing – review & editing, Supervision, Funding acquisition.

## Declaration of Competing Interest

The authors declare that they have no known competing financial interests or personal relationships which have or could be perceived to have influenced the work reported in this article.
